# Ist mehr besser? Politische Implikationen der disparaten Daseinsvorsorge in Deutschland

**DOI:** 10.1007/s41358-020-00239-y

**Published:** 2020-11-03

**Authors:** Matthias Diermeier

**Affiliations:** 1 NRW School of Governance, Universität Duisburg-Essen, Duisburg, Deutschland; 2 Institut der deutschen Wirtschaft, Köln, Deutschland

## Abstract

Die Covid-19-Krise verschärft die bereits lebhaft geführte Diskussion um regionale Disparitäten in der Daseinsvorsorge. Insbesondere im dünnbesiedelten, ländlichen Raum hatten viele Kommunen schon vor der aktuellen Ausnahmesituation Schwierigkeiten, attraktive medizinische Versorgung, Bildungsinfrastruktur, öffentlichen Personennahverkehr oder Breitbandinternet bereitzustellen. Während einerseits die weitere Stärkung urbaner Zentren zur Diskussion steht, wird den Menschen im ländlichen Raum andererseits ein Gefühl des Verlassenwerdens attestiert, das auf einen kulturellen Stadt-Land-Konflikt hinzudeuten scheint. Der vorliegende Beitrag bietet den Auftakt für die kleinräumige empirisch fundierte Debatte um Daseinsvorsorge und die Entfremdung von etablierten Parteien in Deutschland. Die Analyse beruht auf einem zu diesem Zweck aufgebauten Datensatz, der neben einem Index der Daseinsvorsorge auch eine Auswertung der Distanz zum nächstgelegenen Bahnhof und der Erreichbarkeit des nächstgelegenen Krankenhauses auf Gemeindeebene für die Bundestagswahl 2017 sowie die Europawahl 2019 erlaubt. Wie aus anderen westlichen Demokratien zu erwarten, geht auch in Deutschland ein besseres AfD-Ergebnis mit einer schwächeren Daseinsvorsorge einher. Ein Teil dieses Effektes ist jedoch schlicht auf die Divergenz zwischen ländlichem Raum und urbaner Agglomeration zurückzuführen und zeigt sich entgegen der Erwartungen weniger deutlich oder gar nicht zwischen kleineren ländlichen Gemeinden. Zudem steigt die Zustimmung zum Rechtspopulismus weder mit zunehmender Distanz zum nächstgelegenen Bahnhof noch mit einer höheren Kommunalverschuldung. Trotzdem deutet auch in den kleineren Gemeinden einiges auf Erfolge der AfD in Räumen politischer Entfremdung und wirtschaftlicher Schwierigkeiten hin. Robust schneidet die rechtspopulistische Partei umso besser ab desto geringer die vorherrschende Wahlbeteiligung desto stärker die Gemeinde von Abwanderung betroffen sowie desto angespannter der Arbeitsmarkt ist. Eine innovative Wirtschaft vor Ort hat hingegen das Potenzial, die Zustimmung zu den Rechtspopulisten abzumildern.

**Zusatzmaterial online:**

Zusätzliche Informationen sind in der Online-Version dieses Artikels (10.1007/s41358-020-00239-y) enthalten.

## Abstract (en)

The Covid-19 crisis intensifies the lively debate on regional disparities in the supply of public services. Particularly in sparsely populated rural areas, many municipalities had a hard time providing competitive medical care, education facilities, public transport, or broadband internet – even before the Covid-19 outbreak. While on the one hand a further strengthening of urban centres is currently discussed, on the other hand a feeling of being abandoned is perceived among people in rural areas indicating a conflict between agglomerations and the countryside. The present analysis kicks off the empirical debate for Germany: Based on a newly compiled dataset, this contribution provides a spatial analysis, in how far a poor supply of public services is related to an alienation from established political parties in the elections to the Bundestag 2017 and the European elections 2019. An index of public services is created combining amongst others the distance to the closest situated train station as well as the accessibility of the nearest hospital on the municipality level. As expected from other Western democracies, also in Germany a less intense supply of public services is related to a stronger election result for the right-wing AfD. Partly however, this result is simply driven by the stark divergence between rural and urban municipalities and disappears when zooming in on smaller rural municipalities. What is more, AfD support does neither increase with a longer distance to the closest train station nor with a higher level of municipality debt. Nevertheless, the results point to favourable breeding grounds for right-wing populism in areas of political alienation and economic distress. Robustly, the AfD performs better where turnout is lower, where larger shares of the population emigrated and in the face of a tighter labour market. On the contrary, an innovative local economy has the potential to contain right-wing support.

## Einleitung

In East Germany for instance, in the North of Britain, in parts of the US a lot of the complaint has to do with the declining quality of public services. […] The fact that the train station closed, the fact that now I have to go 50 miles to the hospital rather than 10 miles […]. Those lead to feelings of being left behind and feelings of resentment. […] You get a very vicious circle. It may be possible to cut into such a vicious circle by thinking about […] the regional distribution of the resources that people see as crucial for their lives. (Hall 2020, 41:30 min)

Die Implikationen des Siegeszugs attraktiver Großstädte gegenüber dem ländlichen Raum sind in den vergangenen Jahren auch in Deutschland spürbar geworden und werden vermehrt auch von den Regierungsparteien diskutiert (CDU 2018; Fraktionen der CDU/CSU und SPD 2019; Bundesministerium des Inneren, für Bau und Heimat et al. 2019). Während Menschen ländliche Gebiete und Kleinstädte, insbesondere in Ostdeutschland, verlassen, steigt der Migrationsdruck auf die urbanen Agglomerationsgebiete, deren enorme Attraktivität sich aus der Kombination von gut ausgebauter Infrastruktur, attraktivem kulturellen Angebot sowie gut entlohnten Arbeitsplätzen ergibt (Dauth et al. 2018). Aufgrund des zunehmenden Leerstands in den unattraktiven Regionen (Bundesministerium des Inneren, für Bau und Heimat 2018) wird sogar prophezeit, „die Provinz [drohe] zum *Flyover-*Land räumlich Abgehängter zu werden“ (Hervorheb. im Original, Reckwitz 2019, S. 100).

In der Folge dieses Prozesses stehen die weniger urbanen Gebiete vor der Schwierigkeit, angemessene öffentliche Daseinsvorsorge anzubieten und so die in Artikel 72 des Grundgesetzes verankerten „gleichwertigen Lebensverhältnisse“ zu garantieren. Je dünner besiedelt ein Gebiet ist, desto kostenintensiver wird die entsprechende pro Kopf Versorgung mit öffentlichem Personennahverkehr, Bildungsinfrastruktur, Breitbandinternet, sowie mit medizinischem Angebot. Aus rein effizienzbasierten, ökonomischen Überlegungen wird daher die radikale Forderung vorgebracht, Regionalpolitik sollte sich insbesondere auf die urbanen Kerne beschränken und den ländlichen Raum in besonders betroffenen Gebieten sich selbst überlassen (Leibnitz Institut für Wirtschaftsforschung Halle 2019). In eine ähnliche Richtung wird die plakative Äußerung der Bundesforschungsministerin Karliczek interpretiert, „5G ist nicht an jeder Milchkanne notwendig“ – nicht ohne die reflexartige Erwiderung der Gegenseite, dies nehme den entsprechenden Regionen jegliche Entwicklungschancen (Grasnick 2018). Und auch die Kritik an Überkapazitäten und mangelnder Spezialisierung beim öffentlichen Angebot von Krankenhausdienstleistungen zahlt auf den Trade-Off zwischen einer effizienten und einer möglichst breit zugängigen Versorgung ein: Die von der Bertelsmann Stiftung (2019b) vorgebrachte Forderung nach Schließung von ineffizienten Krankenhäusern – insbesondere in ländlichen Regionen – wird nicht zuletzt aufgrund der medizinischen Ausnahmesituation im Zuge von Covid-19 wieder kontrovers diskutiert. Wirtschaftlich verstärkt das Virus zwei entgegenlaufende Trends. Auf der einen Seite wird etwa von Burkhard Jung, dem Präsidenten des Deutschen Städtetags, ein starker lokal verankerter Staat gefordert, der über Daseinsvorsorge Handlungsfähigkeit begründet (Spiegel Online 2020). Dies gilt insbesondere hinsichtlich eines gut ausgebauten Gesundheitssystems, das seine Bürger zu schützen weiß, aber ebenso für das flächendeckende Angebot mit Breitband-Internet, das regionale Disparitäten während des Lock-Downs für Schüler und Arbeitnehmer nicht regionalspezifisch verschärft. Armin Nassehi hofft sogar, dass nach der Krise „staatliche Garantien für Infrastrukturen und die Daseinsvorsorge mit mehr Reputation […] versehen [werden]“ (ZEIT Online 2020). Auf der anderen Seite trifft das Virus einen vulnerablen Akteur besonders hart: die aufgrund der angespannten Finanzlage bereits hoch gefährdeten Kommunen. Der kommunale, Covid-19 bedingte, finanzielle Schaden wird vom Deutschen Landkreistag (2020) für das Jahr 2020 auf 16 Mrd. € taxiert.

Besondere politische Virulenz erhalten die komplexen regionalpolitischen Fragen zudem, weil sie über die ökonomische Divergenz hinaus eng mit einem entsprechenden Stadt-Land Wertekonflikt verknüpft sind. Eindrücklich beschreiben David Goodhart (2017) und Rodríguez-Pose (2018) die geographische Komponente der Spaltung zwischen Globalisten in den Metropolregionen und Lokalisten in der Peripherie, die sich immer weniger durch „das Establishment“ in den Kapitalen vertreten fühlen. Langfristige ökonomische Vernachlässigung in Kombination mit (gefühlter) mangelnder politischer Repräsentation werden als toxische Mischung identifiziert, die sich in Politikverdrossenheit oder starken Wahlergebnissen radikaler rechter Parteien spiegelt („The Revenge of Places that don’t matter“). Aus den USA berichtet Katherine Cramer (2016) vom Gefühl, im ländlichen Wisconsin nicht ausreichend an öffentlichen Ressourcen zu partizipieren, was sie letztlich für die hohe Beliebtheit von Donald Trump mitverantwortlich macht (Guo 2016). Mit Blick auf den Brexit weisen Essletzbichler et al. (2018) darauf hin, dass die Ablehnung der europäischen Union dort am stärksten ausgeprägt ist, wo der Konflikt um knappe öffentliche Ressourcen am intensivsten geführt wird. Und auch für Frankreich haben der Geograph Christophe Guilluy und der Demograph Hervé Le Bras immer wieder medienwirksam den Abstand zum nächsten Bahnhof ins Verhältnis zum Wahlergebnis für den Rassemblement National (vormals Front National) gesetzt (Altwegg 2018; Economist 2017).

Die Konsequenz einer solchen *Geographie der Unzufriedenheit* liegt vermeintlich auf der Hand und wird prominent von Politik und Verwaltung gefordert: Die Stärkung der Daseinsvorsorge im Ländlichen etwa durch die Förderung kommunaler Infrastruktur oder Ausbildungsinstitutionen, um dort wohnhafte Bürger für etablierte Parteien zurückzugewinnen (Süddeutsche Zeitung 2019; Exner 2019; Balser und Braun 2019; Franz et al. 2019). Doch diese Perspektive bleibt nicht unwidersprochen. So erklärt beispielsweise der Economist (2019) die Abwesenheit von Gelbwestenprotesten in der Bundesrepublik mit der dezentralen deutschen Wirtschaftsstruktur, der Innovationskraft – insbesondere der sogenannten „Hidden Champions“ in kleineren Städten – und der Responsivität lokaler Politik auf die Sorgen der Bevölkerung. Die Befunde aus anderen Ländern wären also nicht ohne weiteres auf Deutschland übertragbar.

Eine konsistente empirische Analyse dieser Fragestellungen für Deutschland liegt der Literatur bislang noch nicht vor. Der vorliegende Beitrag hat daher zum Ziel, die Disparitäten der Bereitstellung spezieller öffentlicher Güter zu untersuchen, um den politischen Implikationen des unterschiedlichen Niveaus an Daseinsvorsorge nachzuspüren: Geht eine weniger intensive Versorgung mit öffentlichen Gütern insbesondere im ländlichen deutschen Raum tatsächlich mit einer stärkeren Entfremdung der Bevölkerung von etablierten Parteien einher? Um diese Forschungsfrage empirisch handhabbar zu machen, arbeitet die folgende Analyse die deutschen Spezifika von lokaler Daseinsvorsorge sowie den Zusammenhang zum Aufstieg der rechtspopulistischen Alternative für Deutschland (AfD) heraus und greift auf einen umfangreichen Datensatz auf der kleinstmöglichen räumlichen Ebene zurück.

## Politische Implikationen einer disparaten Daseinsvorsorge

Worauf es hier ankommt, ist die Einsicht, dass sich die Linke durch die Überpolitisierung der kosmopolitischen Konfliktachse in doppelter Hinsicht von den Interessen der sozial Marginalisierten abgewandt hat: einmal durch die Relativierung der Aufmerksamkeit für soziale Problemlagen und dann noch einmal durch die Gegnerschaft zu den „locals“, die umso mehr zunimmt, je mehr der urbane Globalisierungs- und Modernisierungsgewinner zum Zielpunkt politischer Ansprache wird. Die von der kosmopolitischen Party Ausgeschlossenen reagierten bekanntlich in zwei Schüben: zuerst durch Wahlenthaltung und seit einigen Jahren durch vermehrte Hinwendung zum Rechtspopulismus. (Höpner 2018).

Urbane Gewinner und ländliche Verlierer, auch mit Blick auf die Aufmerksamkeit durch das Establishment, so liest sich die Kommentierung des Stadt-Land-Konflikts – wirtschaftlich wie politisch. Daseinsvorsorge, der zugerechnet wird, einst in (West‑)Europa zu nivellierenden Mittelstandsgesellschaften beigetragen zu haben (van Laak 2018), wirkt dann als zusätzliche Zentrifugalkraft, wenn sich die „alte Mittelklasse“ als lokal verankertes „*sesshaftes* Milieu“ (Hervorheb. im Original Reckwitz 2019, S. 98) konstituiert und von ihren demokratisch gewählten Repräsentanten aller politischer Couleur ökonomisch wie politisch im Stich gelassen werden. „Die verwurzelte Existenz der alten Mittelklasse erscheint dann im Verhältnis zur gesellschaftlich geforderten räumlichen Mobilität defizitär“ (Reckwitz 2019, S. 100). Als Konsequenz steht das Abwenden vom etablierten Politikangebot. Die Entscheidung fällt nur noch „zwischen Protest- und Nichtwahl“ (Schäfer 2015, S. 149).

### Gleichwertige Lebensverhältnisse? Daseinsvorsorge in Deutschland

„Daseinsvorsorge mit ihren unterschiedlichen Bereichen gilt in Deutschland als eine wesentliche Grundlage für gleichwertige Lebensverhältnisse“, stellt das Bundesinstitut für Bau‑, Stadt- und Raumforschung (BBSR) (2017, S. 6) fest. Der jüngste Raumordnungsbericht „Daseinsvorsorge sichern“ benennt explizit die Nahversorgung, medizinische Versorgung sowie die Abdeckung mit Bildungseinrichtungen als besonders bedeutsame Teilbereiche (Bundesinstitut für Bau‑, Stadt- und Raumforschung 2017). Erstaunlicherweise sucht man einen konkreten, gesetzlich-verankerten Leistungskatalog vergeblich. Wann der deutsche Staat seiner Verpflichtung zur Daseinsvorsorge nachkommt und wann er sie verfehlt, bleibt demnach notgedrungen „unscharf“ (Dehne 2019, S. 56).

Festgeschrieben hat der Gesetzgeber den Versorgungsauftrag durch den Staat im Raumordnungsgesetz (ROG) § 2 als „Grundsätze der Raumordnung“. In Abs. 1 heißt es: „Im Gesamtraum der Bundesrepublik Deutschland und in seinen Teilräumen sind ausgeglichene soziale, infrastrukturelle, wirtschaftliche, ökologische und kulturelle Verhältnisse anzustreben“. Abs. 3 hält außerdem fest: „Die Versorgung mit Dienstleistungen und Infrastrukturen der Daseinsvorsorge, insbesondere die Erreichbarkeit von Einrichtungen und Angeboten der Grundversorgung für alle Bevölkerungsgruppen, ist zur Sicherung von Chancengerechtigkeit in den Teilräumen in angemessener Weise zu gewährleisten; dies gilt auch in dünn besiedelten Regionen“.

Der zusätzliche Verweis auf die besonders dünn besiedelten Regionen ist als Versicherung des Staates gegenüber all seinen Bürgern und Regionen zu verstehen, diese bei der Bereitstellung eines Mindestmaßes an Grundversorgung gleichermaßen zu bedenken. Die besondere Dringlichkeit des gesetzlich fixierten Versprechens ergibt sich aus der demographischen Entwicklung (Dehne 2019): teure Infrastruktur zu hohen Fixkosten impliziert in Gebieten mit niedriger Bevölkerungsdichte zwangsläufig höhere pro-Kopf-Kosten. Prägnant hält das BBSR die Problematik fest: „Weniger Bevölkerung, weniger Wachstum, weniger Steuern, weniger finanzielle Handlungsmöglichkeiten der öffentlichen Hand“ (Bundesinstitut für Bau‑, Stadt- und Raumforschung 2017, S. 6). Rund die Hälfte der deutschen Gemeinden hat heute eine Einwohnerdichte von unter 100 Personen pro Quadratkilometer.^1^ Gleichwohl gilt auch hier die Verpflichtung zu einer mit den Agglomerationsgebieten gleichwertigen Grundversorgung. Die Abwanderung ganzer Bevölkerungskohorten stellt in diesem Kontext insbesondere ländliche, ostdeutsche Regionen vor große Herausforderungen (Oberst et al. 2019). Demographische Prognosen lassen für die Zukunft sogar auf eine Verschärfung des Problems schließen (Bundesinstitut für Bau‑, Stadt- und Raumforschung 2015). Das in § 2 ROG Abs. 4 festgeschriebene Ziel, „den Raum im Hinblick auf eine langfristig wettbewerbsfähige und räumlich ausgewogene Wirtschaftsstruktur und wirtschaftsnahe Infrastruktur sowie auf ein ausreichendes und vielfältiges Angebot an Arbeits- und Ausbildungsplätzen zu entwickeln“, rückt damit in weite Ferne. Ländliche Regionen, die lange Zeit mit einem stabilen Arbeitsangebot insbesondere in Ausbildungsgeberufen um Unternehmensansiedlungen werben konnten, verlieren ohne das entsprechende Humankapital im Standortwettbewerb an Wettbewerbsfähigkeit. Die niedrigeren Löhne im Ländlichen bedingen dann eine steigende Einkommensungleichheit zwischen Stadt und Land (Dauth et al. 2018). Eine entsprechend geringere wirtschaftliche Aktivität vor Ort führt zu geringeren Steuereinnahmen und bringt besonders die kommunale Ebene in die Defensive, die nicht nur mit steigenden pro-Kopf-Kosten sondern ebenso mit sinkenden Einnahmen zu kämpfen hat (Heinrich-Böll-Stiftung 2017). Die Strahlkraft einer attraktiven Daseinsvorsorge geht damit weit über die individuelle Dimension hinaus und erhält eine regionalpolitische Bedeutung.

Grundsätzlich weist die kommunale Selbstverwaltungsgarantie (§ 28 Abs. 2 Satz 1 GG) den Kommunen das Recht und die Verantwortung zu, der kommunalen Daseinsvorsorge (bspw. mit ÖPNV, medizinischer Versorgung oder Bildungsangeboten) mit dem Ziel gleichwertiger Lebensverhältnisse nachzukommen (Schuppli 2017; Kahl und Lorenzen 2019). Um diesem Anforderungsportfolio gerecht zu werden und gleichzeitig den weiteren Verpflichtungen – wie beispielsweise die während einer Wirtschaftskrise stark ansteigenden Kosten der Unterkunft für Leistungsbeziehende nach SGBII – nachzukommen, haben Kommunen das grundgesetzlich verankerte Recht auf finanzielle Mindestausstattung. Selbstverschuldet oder aufgrund von Migration und Strukturwandel steckten einige Gemeinden jedoch bereits vor der Covid-19-Pandemie in einer „kommunalen Finanzkrise“ (Beznoska und Kauder 2019, S. 1). Insbesondere Kommunen in Nordrhein-Westfalen, dem Saarland sowie Rheinland-Pfalz sind dazu übergegangen, strukturell Ausgaben mit Kassenkrediten zu finanzieren, die als Instrument eigentlich nur für den Liquiditätsausgleich vorgesehen sind. Eine entsprechend geringere Investitionstätigkeit weisen betroffene Gemeinden auf. Zudem geht ein hoher Bestand an Kassenkrediten mit hohen Grund- und Gewerbesteuersätzen einher – der Haupteinnahmequelle für Gemeinden. Eine Erhöhung der Hebesätze mag zwar kurzfristig die kommunale Haushaltslage befrieden, macht den Standort mittelfristig für Unternehmensansiedlungen aber unattraktiv (Beznoska und Hentze 2019). Die aktuelle Situation verschärft die Gefahr, dass Kommunen in einen Teufelskreis hineinrutschen, in dem sie aus strukturellen wirtschaftlichen Problemen heraus die Unternehmenssteuerlast erhöhen, um dem negativen Haushaltssaldo Herr zu werden. Damit werden sie aber noch unattraktiver und verzeichnen letztlich eine noch geringere wirtschaftliche Aktivität sowie geringere Steuereinnahmen, mit denen sie den Aufgaben der Daseinsvorsorge kaum noch gerecht werden können. Selbst ein innovativer und integrativer Ansatz der Daseinsvorsorge, der staatliche Akteure entlastet, indem er die Zivilgesellschaft sowie lokale Genossenschaften mit einbindet, läuft ohne kommunale Handlungsfähigkeit und Infrastruktur ins Leere (Heinze 2020, S. 166).

Tatsächlich deutet der kommunale Finanzreport der Bertelsmann Stiftung auf eine Divergenz der Kommunalfinanzen hin: „Die Schere zwischen starken und schwachen Kommunen öffnet sich seit vielen Jahren. Die Lebensverhältnisse der Menschen sind mehr und mehr abhängig von ihren Wohnorten. Messbar wird dies unter anderem an den Haushaltskennzahlen“ (Bertelsmann Stiftung 2019a, S. 4).^2^ Wirtschaftliche Aktivität, der Arbeitsmarkt, die kommunale Bereitstellung öffentlicher Güter sowie die Lebensqualität sind demnach eng verknüpft. Der Staat mag in prosperierenden Regionen stark und durchsetzungsfähig sein, sich in wirtschaftlich schwächeren Regionen hingegen zurückgezogen haben. Einem solchen Befund stellen Fuest und Immel (2019) entgegen, dass die Regionen sich in Deutschland etwa mit Blick auf die verfügbaren Haushaltseinkommen angleichen und keinesfalls weiter auseinanderdriften. Dabei erscheint möglich, dass das Niveau der kommunalen Daseinsvorsorge abhängiger vom Wohnort wird, die individuelle Einkommensperspektive hiervon aber nicht grundsätzlich betroffen sein muss.

Eine marode Grundversorgung durch einen schwachen Staat, kann dann – auch ohne Veränderung der eigenen finanziellen Ausstattung – ein Gefühl des Verlassen- oder Abgehängtseins induzieren, aus dem heraus sich politische Frustration nährt. Politische Frustration kann sich mit Blick auf die Daseinsvorsorge zusätzlich auch aus einer *gefühlten* und nicht unbedingt aus einer *tatsächlichen* Benachteiligung herausbilden. Welche Leistungen vom Gesetzgeber formal unter die Daseinsvorsorge gefasst werden, ist dafür grundsätzlich irrelevant. So kann beispielsweise eine Schulschließung oder das Abschaffen einer Nahverkehrsanbindung von den Betroffenen aufgrund der Sichtbarkeit als ein „Verlassenwerden“ eingeordnet werden. Die Heinrich-Böll-Stiftung (2017) hebt hervor, dass es den Bürgern bei der Evaluation von Daseinsvorsorge mehr auf „Outcomes“ als auf reine Inputfaktoren ankommt. Wie gut oder schlecht beispielsweise das kommunale Bildungsangebot bewertet wird, muss nicht zwangsläufig in einem Zusammenhang mit der Anzahl an Schulplätzen innerhalb einer Gemeinde stehen, sondern hänge vielmehr von der *Qualität* des schulischen Angebots im Ganzen ab. Ein ähnliches Argument führt die Bertelsmann Stiftung (2019b) an, wenn sie für eine bessere medizinische Versorgung eine Stärkung von Spezialkliniken auf Kosten der „Zahl der Standorte“ fordert.

Nicht zuletzt aus dieser Abwägungen von Effizienz- gegenüber Distributionsargumenten lässt sich der radikale Vorschlag vom Leibnitz Institut für Wirtschaftsforschung Halle (2019) einordnen, Ausgaben für (mittlerweile) dünnbesiedelte Regionen in Ostdeutschland drastisch zurückzufahren und auf eine Stärkung der Zentren zu setzen. Dem muss entgegengehalten werden, wie symbolträchtig der Rückzug des Staates im ländlichen Raum sowie der (qualitative) Aufbau von Kapazitäten in den Zentren empfunden werden kann. Selbst wenn die Bündelung der Dienstleistungen letztlich die Angebote auf Kosten einer größeren Entfernung in den abgelegenen Kommunen qualitativ verbessert, muss dies schlussendlich von den Bürgern nicht so empfunden werden.

### Der schwache Staat – politische Entfremdung aufgrund mangelnder Daseinsvorsorge?

Fühlen sich Wählerinnen und Wähler etwa mit Blick auf die Bereitstellung öffentlicher Güter benachteiligt, besteht die Gefahr eines Verfallens in „Apathie und Gleichgültigkeit“ (Bertelsmann Stiftung 2013b, S. 4), woraufhin die „Exit“ Strategie, bei der das Establishment durch ein Fernbleiben von der Wahlurne abgestraft wird, an Attraktivität gewinnt (Pickel 2019). Empirische Untersuchungen der Wahlbeteiligung bekräftigen diese Interpretation. So finden Haußner und Kaeding (2019) sowie Schäfer und Roßteutscher (2015) in ihren Analysen der Bundestagswahlen in 2017 und 2013 mit Blick auf die Wahlbeteiligung einen klaren Zusammenhang zur regionalen sozio-ökonomischen Lage: in wirtschaftlich benachteiligten Gebieten zieht es weniger Menschen an die Wahlurne als in der prosperierenden Agglomeration. Ähnliches gilt innerhalb von Städten. Einhellig wird die bedenkliche sozio-ökonomische Spaltung der politischen Partizipation herausgearbeitet, bei der wirtschaftlich schwächere Schichten eine wesentlich geringere Wahlbeteiligung aufweisen:^3^ Kaeding et al. (2016, S. 14) nennen diese Beobachtung „die soziale Schieflage der niedrigen Wahlbeteiligung“. Schäfer (2015) erkennt einen „Verlust politischer Gleichheit“. Und die Bertelsmann Stiftung (2013a) konstatiert sogar eine „gespaltene Demokratie“.

Umgekehrt hat sich der Trend der fallenden Wahlbeteiligung erst mit der gestiegenen Partizipation bei der Bundestagswahl 2017 und der Europawahl 2019. Als besonders bedeutsam wird dafür der Erfolg der rechtspopulistischen AfD eingeschätzt: Die Bertelsmann Stiftung (2017) spricht explizit von einem „AfD-Effekt“, der die soziale Spaltung der Wahlbeteiligung gebremst habe. Auch mit Blick auf die Wahl zum Europäischen Parlament schneidet die AfD besonders stark in wirtschaftlich und demographisch gefährdeten Kreisen ab (Franz et al. 2019). Pickel (2019) bescheinigt Wählerinnen und Wählern, die sich vormals aus dem politischen System qua Wahlenthaltung zurückgezogen hatten („Exit“), nun mit einer Protestwahl („Voice“) auf sich aufmerksam gemacht zu haben. Von einer „Revolte an der Wahlurne“ (Rodríguez-Pose 2018) ist zwar nicht die Rede, aber tatsächlich hat sich die AfD 2017 in den Wahlkreisen erfolgreich gezeigt, in denen die Wahlbeteiligung von besonders niedrigem Niveau stark angestiegen ist (Haußner und Leininger 2018).

Obwohl durchaus bezweifelt werden darf, dass sich die deutschen Rechtspopulisten für mehr Umverteilung und eine stärkere Daseinsvorsoge einsetzen würden (Diermeier 2020) und die etablierten Parteien durch Gegenmobilisierung ebenfalls zu einem Anstieg der Wahlbeteiligung beigetragen haben (Bertelsmann Stiftung 2017; Franz et al. 2019), zeigt sich innerhalb der AfD-Anhängerschaft doch ein hohes Maß an Enttäuschung über das politische Establishment (Bieber et al. 2018)^4^ sowie Anzeichen für Erfolge im wirtschaftlich schwachen ländlichen Raum (Franz et al. 2018, 2019).^5^ Entgegen der vielfach geäußerten Stadt-Land-Spaltung als Erklärungsmuster für die Wahlerfolge der AfD (Franz et al. 2018; Hillje 2018), stellen Deppisch et al. (2019) klar, dass die starken Ergebnisse der Partei bei der Bundestagswahl 2017 keinesfalls über einen simplifizierten Stadt-Land-Konflikt erklärt werden können. Zwar schneidet die AfD in Ostdeutschland tatsächlich in häufig prekären, eher ländlichen Gebieten gut ab, für Westdeutschland, wo einige ländliche Regionen wirtschaftlich prosperieren, birgt die Differenzierung hingegen keinen Erklärungsgehalt.

Benedikt Kaiser (2019), ein Autor der Neuen Rechten, vermutet in seinem „Blick nach Links“ (Antaios Verlag), die Entfremdung zwischen Wählerschaft und etablierten Parteien habe wirtschaftliche wie kulturelle Wurzeln, die sich letztlich in Fragen der Daseinsvorsorge spiegelten: „Die berechtigten Sorgen um Kindergartenplätze, Alltagssicherheit und soziale Fürsorgesysteme, die für kosmopolitische Wohlstandslinke banale, zu ignorierende Themen sind, und die sich auch und vor allem aufgrund der ungehemmten Zuwanderung verschärften, werden also mit dem Verdikt ‚Rassismus‘ kontaminiert, anstatt sie lösungsorientiert in den Fokus zu stellen“ (2019, S. 30). Und tatsächlich wird auf der Suche nach treffsichereren Erklärungsmustern für die AfD-Erfolge im Ländlichen häufig ein Zusammenhang zur öffentlicher Daseinsvorsorge suggeriert. Solche Befunde beruhen jedoch auf anekdotischer Evidenz und nicht auf einer konsistenten empirischen Analyse. So beschreibt etwa Bednarz (2017) den Rückzug des Staatlichen aus dem ländlichen Raum durch das Schließen zentraler Orte des Austausches, zu denen sie auch Apotheken^6^ und Arztpraxen zählt.^7^ Den Erfolg der Rechtspopulisten will sie als Mahnung an die etablierten Parteien verstanden wissen, „den ländlichen Raum nicht nur in ihren Wahlprogrammen, sondern auch vor Ort nicht zu vernachlässigen“. Vergleichbare Schlussfolgerungen zieht Hillje (2018) aus 500 „Haustürgesprächen“ in wirtschaftlich gefährdeten Regionen Deutschlands und Frankreichs, in denen die rechtspopulistischen Parteien besonders gut abgeschnitten haben: Die Befragten nennen „Lücken der Daseinsvorsorge“ (Hillje 2018, S. 15) wie die lokale Verkehrs- und Sozialinfrastruktur am häufigsten als größtes Problem am Wohnort.

Kurtenbach (2019) systematisiert die teils widersprüchlichen Studien zur räumlichen Struktur des Rechtspopulismus und bietet einen hilfreichen Überblick über die Erklärungshypothesen. Mit Blick auf das Angebot öffentlicher Güter bietet die *Marginalisierungshypothese *einen wichtigen Ansatzpunkt. Diese postuliert neben der generellen wirtschaftlichen Perspektive eine besondere Bedeutung an bereitgestellten öffentlichen Gütern. Hierunter lässt sich etwa die mögliche politische Entfremdung aufgrund einer großen Distanz zum nächstgelegenen Bahnhof subsumieren, die durch Hevré Le Bras und Christophe Guilluy angeführt wird (Economist 2017; Altwegg 2018). Guilluy betont zudem das Bedürfnis nach verstärkter Bereitstellung öffentlicher Daseinsvorsorge in politisch-entfremdeten und wirtschaftlich-gefährdeten Regionen: „Dans cette France populaire, la critique de la mondialisation économique se combine avec une défense d’un État protecteur et des services publics“ (2011, S. 44). Dabei verschwimmen die auf den ersten Blick *klaren* wirtschaftlichen mit *komplexen *kulturellen Erklärungsmustern: Denn mit dem Wunsch nach einem präsenteren Staat, der sich nach Meinung von Guilluy und Le Bras insbesondere aus dem peri-urbanen Raum zurückgezogen hat, geht auch ein gefühltes Verlassenwerden durch eine als immer entfernter empfundene Elite in den metropolitanen Zentren einher (Guilluy 2018; Le Bras 2015). Für die Zustimmung zu einem EU-Austritt beim Brexit-Referendum liefert Fetzer (2019) ein vergleichbares Argument: Während der Euro-Krise verzeichneten einige britische Distrikte einen Rückgang der Sozialstaatsausgaben um bis zu 50 %. Von den Kürzungen Betroffene gaben in der Folge an, sie hätten keinen Einfluss mehr auf die Politik oder die politischen Repräsentanten kümmerten sich nicht ausreichend. Der Rückzug des Staates in den entsprechenden Regionen lässt sich schließlich als „Austeritäts-Effekt“ für den Ausgang des Brexit-Referendums mitverantwortlich machen.^8^

In seiner Gesamtschau deutet der vorliegende Literaturüberblick darauf hin, dass die rechtspopulistischen Erfolge in Deutschland – vergleichbar mit anderen Ländern – im Sinne der Marginalisierungshypothese mit regionalen Disparitäten der sozio-demographischen Lage im Allgemeinen und dem Angebot von kommunaler Daseinsvorsorge im Speziellen in Verbindung stehen. Im Kontext des Stadt-Land-Konfliktes liegt die Vermutung nahe, dass weniger die Stadt-Land-Spaltung per se eine Entfremdung von etablierten Parteien im Ländlichen bedingt, sondern der Zusammenhang vielmehr moderiert wird, durch das teilweise sehr schwache Angebot mit öffentlichen Gütern im ländlichen Raum.

#### Hypothese 1

Die AfD-Wahlergebnisse fallen je besser aus, desto schwächer eine Region mit Gütern der öffentlichen Daseinsvorsorge ausgestattet ist.

#### Hypothese 2

Besonders deutlich zeigt sich der Zusammenhang zwischen AfD-Wahlergebnis und Daseinsvorsorge in kleineren Kommunen, die kaum ein attraktives Niveau an öffentlichen Gütern bereitstellen können.

## Datenmaterial und methodisches Vorgehen

Häufig verharren kleinteilige räumliche Studien zum Wahlverhalten auf Stadtteilebene und klammern aus Gründen der Datenverfügbarkeit den ländlichen Raum als Untersuchungsgegenstand aus (Kurtenbach 2019; Haußner und Kaeding 2019; Schäfer 2015; Gardemin 2009; Geiling 2009). Alternativ werden Wahlergebnisse auf der aggregierten Ebene von Landkreisen untersucht, die kaum die hohe Diversität der kleineren Gemeinden abzubilden vermögen (Bergmann et al. 2018; Franz et al. 2018, 2019). Die vorliegende Analyse bemüht sich daher um eine deutschlandweite Datengrundlage auf Gemeindeebene, die sowohl ländlichen Raum als auch Agglomerationsgebiete abzudecken vermag. Eine solche Operationalisierung geht auf Kosten von Datenverfügbarkeit einerseits sowie Trennschärfe des Wahlverhaltens innerhalb der großen Agglomerationsräume andererseits. Wie Leggewie (2019) zu Recht kritisiert, vermag dieserart Analyse insbesondere die unterschiedliche Lebenswirklichkeit zwischen eng beieinanderliegenden Quartieren nicht abzubilden. Weniger treffend zeigt sich dieses Argument gegenüber der relativ homogenen Erreichbarkeit von öffentlicher Infrastruktur innerhalb der großen Städte, wo sich selbst die in den 1960er-Jahre errichteten „Satellitenstädte“ durch eine gute Zentrumsanbindung auszeichnen (Friedrich 1977). Zudem ermöglicht eine Analyse auf Gemeindeebene aufgrund der Vielzahl an Kommunen einen besonderen Fokus auf den ländlichen Raum.

Als Proxy der Entfremdung von etablierten Parteien in Deutschland wird in der folgenden empirischen Analyse das AfD-Wahlergebnis der Bundestagswahl 2017 (Zweitstimmenergebnis) sowie der Europawahl 2019 auf Gemeindeebene („Voice“) als abhängige Variable genutzt. Entgegen möglicherweise verzerrter Befragungsdaten haben amtliche Wahlstatistiken grundsätzlich den Vorteil, die tatsächlichen Wahlergebnisse abzubilden (Schwander und Manow 2017; Manow 2018). Als Datengrundlage werden daher die entsprechenden vom Bundeswahlleiter bereitgestellten Wahlergebnisse auf Wahlbezirksebene herangezogen.^9^ Diese lassen sich von Wahlbezirks- auf Gemeindeebene hochaggregieren. Teilen sich mehrere Wahlbezirke einen Briefwahlbezirk über Gemeindegrenzen hinweg, lassen sich die Briefwähler jedoch nicht eindeutig einem Wahlbezirk zuordnen. Anstatt diese Briefwahlbezirke zu ignorieren, werden die Stimmen des gemeinsamen Briefwahlbezirks nach dem Anteil der jeweilig abgegebenen Urnenwahlstimmen an allen abgegeben Urnenwahlstimmen der zugehörigen Wahlbezirke verteilt. Die hieraus berechnete Wahlbeteiligung („Exit“) wird als Kontrollvariable in die Regression aufgenommen.^10^

**Figure 1:**
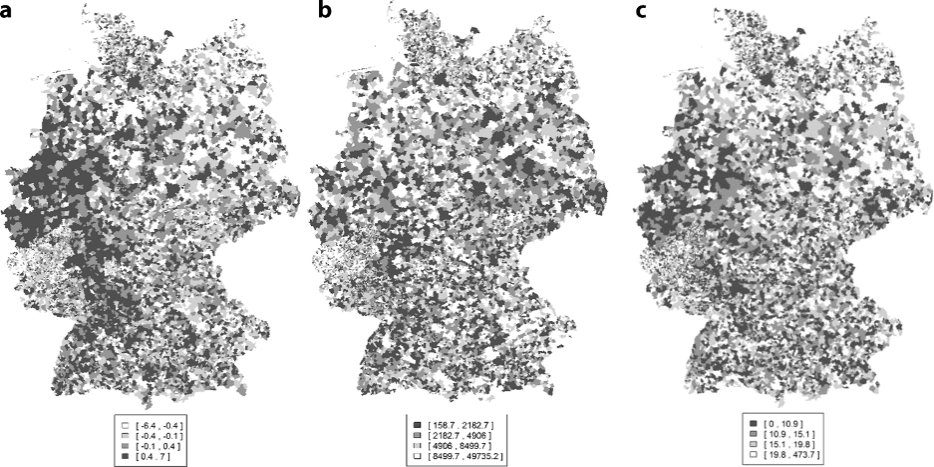

Als Dimension der öffentlichen Daseinsvorsorge werden das medizinische Angebot, die Verkehrsinfrastruktur, die digitale Infrastruktur sowie die Bildungsinfrastruktur betrachtet.^11^ Wie in § 2 ROG Abs. 3 festgeschrieben, ist in den einzelnen Dimensionen besonders die „Erreichbarkeit von Einrichtungen und Angeboten der Grundversorgung“ von Bedeutung.^12^ Bezüglich der *medizinischen Infrastruktur* wird auf den Anteil der Haushalte, die in einem Kilometer Umkreis einer Apotheken leben, aus der INKAR-Datenbank sowie auf die Erreichbarkeit von Krankenhäusern in Pkw-Fahrminuten, bereitgestellt vom GKV-Kliniksimulator, zurückgegriffen.^13^ Aufgrund der durch Covid-19 neu angestoßenen Diskussion um Daseinsvorsorge mit Krankenhausinfrastruktur werden alle Regressionen mit der Erreichbarkeit des nächstgelegensten Krankenhauses als separate unabhängige Variable berechnet. Als Proxy der *Verkehrsinfrastruktur* werden die Erreichbarkeiten von Autobahnen in Pkw-Fahrminuten sowie die Abfahrten des ÖPNV pro Kopf aus der INKAR-Datenbank genutzt. Die Haltestellen des ÖPNV werden nicht in Erreichbarkeit ausgelesen, da diese eine zu geringe Varianz aufweisen. Insbesondere in dünn besiedelten Gebieten ist nicht nur bedeutsam, ob es etwa eine Busanbindung gibt, sondern wie diese getaktet ist. Um den Zusammenhang zwischen geographischem Abstand zum nächstliegenden Bahnhof und AfD-Wahlergebnis gesondert zu testen, wird dieser für alle 5500 Bahnhöfe der Deutschen Bahn und je Kommune als durchschnittliche bevölkerungsgewichtete Distanz berechnet.^14^ Als *digitale Daseinsvorsorge* wird auf den Anteil an Haushalten innerhalb einer Kommune, der von einer Breitbandversorgung mit 100Mbit abgedeckt ist, aus der INKAR-Datenbank zurückgegriffen. Die *Bildungsinfrastruktur* wird durch den Anteil von Schülern in allgemeinbildenden Schulen an der Bevölkerung zwischen 6 und 18 Jahren aus der INKAR-Datenbank abgedeckt.^15^ Die Variablen der kommunalen Daseinsvorsorge werden standardisiert und gleichgewichtet zu Indices der medizinischen, digitalen, verkehrs- und bildungsinfrastrukturellen Daseinsvorsorge, und dann zu einem Gesamtindex dieser vier Kategorien aggregiert. Hierfür werden Variablen, die in Erreichbarkeiten gemessen werden, negativ kodiert, sodass eine hohe Ausprägung der Indices eine bessere Versorgung mit Daseinsvorsorge widerspiegelt. Abb. 1 zeigt die räumliche Ausprägung des Gesamtindex und beispielhaft die aus der aktuellen Diskussion als besonders relevant charakterisierten Variablen Distanz zum nächstgelegenen Bahnhof sowie Erreichbarkeit des nächstgelegenen Krankenhaus.

Da nur ein Teil der hier betrachteten Variablen der kommunalen Verantwortung obliegt, wird diese Dimension der Analyse zusätzlich durch die Kommunalverschuldung abgebildet, die von den Statistischen Bundesämtern des Bundes und der Länder bereitgestellt wird.^16^ Schließlich schränkt die häufig persistente und hohe Verschuldung die kommunale Handlungsfähigkeit auch mit Blick auf das Erbringen von Daseinsvorsorge vor Ort ein (Beznoska und Kauder 2019). Als sozio-ökonomische Kontrollvariablen auf Gemeindeebene muss aufgrund der schwierigen Datenverfügbarkeit in erster Linie auf Daten der Bundesagentur für Arbeit (BA) Bezug genommen werden. Diese stellt den Anteil an Arbeitslosen (SGB II und SGB III) und der erwerbsfähigen Bevölkerung zur Verfügung – nach Haußner und Kaeding (2019) der bedeutsamste Indikator, der die soziale Lage in Wahlanalysen abbildet.^17^ Aus der Datenbank der BA lässt sich zudem der Anteil der Erwerbstätigen im Verarbeitenden Gewerbe berechnen, der sich in vergangenen Regionalanalysen als wichtiger Prädiktor des AfD-Wahlergebnis erwiesen hat (Bergmann et al. 2018; Manow 2018).^18^ Besondere Bedeutung kommt in der vorliegenden Analyse der IW-Patentdatenbank (Koppel und Röben 2019) zu. Diese umfasst eine Georeferenzierung aller Patenterstanmeldungen deutscher Anmelder beim deutschen Patent- und Markenamt (DPMA) nach Anmeldersitz. Die Patenttätigkeit auf kommunaler Ebene bietet im Gegensatz zu den schlichten Beschäftigungsanteilen in der Industrie einen Proxy für die Innovationskraft und Zukunftsträchtigkeit der lokal ansässigen Wirtschaft. Als wichtige Variable im Stadt-Land-Kontext wird die Bevölkerungsdichte auf Gemeindeebene der INKAR-Datenbank entnommen.^19^ Der Altenquotient (Anteil der Bevölkerung älter als 65 Jahre) bildet die Demographie aus den Daten der Statistischen Bundesämtern des Bundes und der Länder ab.^20^

Nicht alle Datenquellen können für alle Gemeinden die gewünschten Informationen bereitstellen. Bei vielen der Kommunen, für die Daten nicht vollständig zusammengetragen werden konnten, handelt es sich um kleine Gemeinden. Wo dies möglich ist, werden die fehlenden Werte mit den entsprechenden Durchschnitten der Landkreise imputiert.^21^ Für die wenigen Gemeinden, in denen ein solches Vorgehen nicht möglich ist, werden die fehlenden Werte mithilfe der vorhandenen Variablen durch multiple Imputation geschätzt, sodass die Datenbasis 11.049 Gemeinden im Jahr 2017 in Deutschland umfasst. Um zu verhindern, dass Großstädte wie Berlin oder Köln und Kleinstgemeinden wie die in Schleswig-Holstein gelegenen Insel-Gemeinde Gröde (9 Einwohner) oder das rheinland-pfälzischen Hamm (17 Einwohner) mit gleicher Gewichtung in die Regression eingehen, wird in den Regressionen eine Bevölkerungsgewichtung vorgenommen. Damit die Ergebnisse aber nicht ausschließlich durch die 80 Großstädte mit mehr als 100.000 Einwohner und die 618 Mittelstädte mit zwischen 20.000 und 100.000 Einwohnern determiniert werden, werden alle Regressionen auch exklusiv für die 10.350 Gemeinden mit weniger als 20.000 Einwohnern sowie für alle 8120 Gemeinden mit weniger als 5000 Einwohnern gerechnet und ausgewiesen. Dieses Vorgehen soll einen besonderen Analysefokus auf die häufig vernachlässigten kleinen Kommunen im ländlichen Raum lenken.

Zudem ist die Datenverfügbarkeit am aktuellen Rand eingeschränkt. Von allen Kontrollvariablen steht für das Jahr 2019 lediglich die Arbeitslosenquote zur Verfügung, sodass für die Regressionen der Europawahl 2019 für alle weiteren Variablen auf die Datenbasis 2017 zurückgegriffen werden muss. Von einer Längsschnittanalyse muss aufgrund der mangelnden Datenverfügbarkeit der Daseinsvorsorge-Variablen in der Vergangenheit abgesehen werden. Eine solche dynamische Analyse, die etwa den Einfluss der Schließung von Apotheken und Bahnhöfen oder den Anschluss an das Breitbandnetz auf das Wahlergebnis beleuchtet, hat mit einer breiteren Datenbasis in Zukunft das Potenzial, sich kausalen Schlussfolgerungen anzunähern. Der vorliegende Querschnitt, der mithilfe von OLS-Regressionen geschätzt wird, ist lediglich als vertiefende Korrelationsanalyse zu betrachten, bietet aber nichtsdestotrotz wichtige erste Anhaltspunkte, inwiefern Daseinsvorsorge und das rechtspopulistische Wahlergebnis in Deutschland miteinander einhergehen.^22^ Den strukturellen regionalen Unterschieden wird mit Bundeslanddummies Rechnung getragen.^23^

## Räumliche Determinanten des AfD-Wahlergebnis

Bei der Bundestagswahl 2017 schaffte mit der AfD erstmalig eine rechtspopulistische Partei in Fraktionsstärke den Sprung in den deutschen Bundestag. Im Vergleich mit der Bundestagswahl 2013 ging der Erfolg der Rechtspopulisten mit einem Anstieg der Wahlbeteiligung um 4,7 Prozentpunkte einher. Das starke AfD-Ergebnis von 12,7 % im Jahr 2017 konnte bei der Europawahl entgegen der hohen Erwartungen mit lediglich 11 % nicht eingestellt werden, dabei stieg die Wahlbeteiligung bei der Europawahl gegenüber 2014 sogar um 13,2 Prozentpunkte an. Dies lag wohl auch daran, dass 2019 neben der Mobilisierung durch die AfD auch eine besonders starke Gegenmobilisierung durch den politischen Antagonisten, Bündnis 90/Die Grünen, erfolgte (Franz et al. 2019).

### Bundestagswahl 2017

Tab. 1 zeigt die OLS-Regressionsoutputs mit dem AfD-Zweitstimmenanteil der Bundestagswahl 2017 als abhängige Variable sowie dem Index der Daseinsvorsorge und seinen Subindices als unabhängige Variablen. Zudem werden die Regressionsergebnisse mit der prominent diskutierten Distanz zum nächstgelegenen Bahnhof und der Erreichbarkeit des nächstgelegenen Krankenhauses als unabhängige Variablen separat ausgewiesen.

**Table 1:** 

	(1)	(2)	(3)	(4)	(5)	(6)
	AfD Ergebnis	AfD Ergebnis <20.000	AfD Ergebnis	AfD Ergebnis <20.000	AfD Ergebnis	AfD Ergebnis <20.000
Daseinsvorsorge Index	−0,014*** (0,001)	−0,008*** (0,001)	–	–	–	–
Daseinsvorsorge Mobilität	–	–	0,001 (0,001)	0,001 (0,001)	–	–
Daseinsvorsorge Digital	–	–	−0,002*** (0,000)	−0,000 (0,000)	–	–
Daseinsvorsorge Gesundheit	–	–	−0,009*** (0,001)	−0,006*** (0,001)	–	–
Daseinsvorsorge Bildung	–	–	−0,006*** (0,000)	−0,003*** (0,000)	–	–
Distanz Bahnhof	–	–	–	–	−0,000 (0,000)	−0,000 (0,000)
Erreichbarkeit Krankenhaus	–	–	–	–	0,005*** (0,000)	0,001*** (0,000)
Wahlbeteiligung	−0,191*** (0,008)	−0,155*** (0,008)	−0,201*** (0,008)	−0,168*** (0,008)	−0,165*** (0,008)	−0,140*** (0,008)
Anteil Beschäftigung Verarbeitendes Gewerbe	0,019*** (0,002)	0,005*** (0,002)	0,022*** (0,002)	0,007*** (0,002)	0,020*** (0,002)	0,005*** (0,002)
Einwohnerdichte	−0,003** (0,001)	0,005* (0,002)	−0,001 (0,001)	0,006** (0,003)	−0,009*** (0,001)	−0,007*** (0,002)
Einwohnerdichte^2	−0,000*** (0,000)	−0,000*** (0,000)	−0,000*** (0,000)	−0,000*** (0,000)	0,000 (0,000)	0,000 (0,000)
Anteil Arbeitslose	0,483*** (0,024)	0,322*** (0,027)	0,471*** (0,024)	0,320*** (0,027)	0,458*** (0,025)	0,285*** (0,028)
Veränderung Anteil Arbeitslose	−0,014*** (0,002)	−0,013*** (0,002)	−0,014*** (0,002)	−0,013*** (0,002)	−0,015*** (0,002)	−0,013*** (0,002)
Patentanmeldungen pro Kopf	−0,260** (0,121)	−0,611** (0,288)	−0,297** (0,120)	−0,606** (0,287)	−0,421*** (0,123)	−0,769*** (0,290)
Altenquotient	0,080*** (0,009)	−0,089*** (0,009)	0,094*** (0,009)	−0,080*** (0,009)	0,052*** (0,009)	−0,099*** (0,009)
Bevölkerungsentwicklung	−0,460*** (0,086)	−0,104 (0,077)	−0,422*** (0,086)	−0,091 (0,077)	−0,773*** (0,087)	−0,235*** (0,077)
Kommunalverschuldung	−0,320** (0,150)	−1,082*** (0,243)	−0,484*** (0,151)	−1,140*** (0,242)	−0,392** (0,152)	−1,356*** (0,244)
Bundesland fixe Effekte	✔	✔	✔	✔	✔	✔
Konstante	0,190*** (0,008)	0,218*** (0,008)	0,195*** (0,008)	0,225*** (0,008)	0,177*** (0,008)	0,211*** (0,008)
*N*	11.049	10.350	11.049	10.350	11.049	10.350
R‑Quadrat	0,798	0,796	0,800	0,797	0,791	0,793

Standardfehler in Klammern

*** *p* < 0,01; ** *p* < 0,05; * *p* < 0,1

Die OLS-Regressionsergebnisse mit dem AfD-Wahlergebnis der Bundestagswahl 2017 als abhängige Variable in Tab. 1 bestätigen wie in Hypothese 1 erwartet, dass grundsätzlich eine bessere Daseinsvorsorge ceteris paribus statistisch signifikant mit einem schlechteren AfD-Wahlergebnis auf kommunaler Ebene einhergeht. Eine Gemeinde mit einer um eine Standardabweichung intensiveren Daseinsvorsorge (Gesamtindex) geht mit einem um 0,14 Standardabweichungen schlechteren AfD-Wahlergebnis einher – lediglich Wahlbeteiligung und Arbeitslosigkeit weisen einen höheren Beta-Koeffizienten aus.^24^ Entgegen Hypothese 2 verringert die Reduktion des Samples auf Gemeinden mit weniger als 20.000 Einwohnern die Effektstärke jedoch deutlich auf 0,07 Standabweichungen. Dieses Muster spiegeln auch die Regressionsergebnisse der Subindices – der Effekt des Breitbandversorgungsindex verliert sogar seine statistische Signifikanz im reduzierten Sample. Der Subindex zur Daseinsvorsorge mit Mobilitätsinfrastruktur weist zudem überhaupt keine statistische Signifikanz auf, ebenso wie die durchschnittliche Distanz innerhalb einer Gemeinde zum nächstgelegenen Bahnhof, dem für die rechtspopulistischen Wahlerfolge in Frankreich eine hohe Bedeutung zugemessen wird (Economist 2017). Der Effekt zwischen AfD-Ergebnis und Erreichbarkeit des nächsten Krankenhauses ist hingegen statistisch signifikant. Auch hier sinkt jedoch der Beta-Koeffizient mit der Reduktion des Samples deutlich von 0,05 auf 0,02 Standardabweichungen.^25^ Zum einen weisen die schwächeren Effekte innerhalb der Kleinstädte und dem ländlichen Raum darauf hin, dass Unterschiede im AfD-Wahlergebnis zu einem Großteil mit der Disparität zu den gut versorgten Agglomerationsgebieten in Verbindung stehen. Zum anderen lassen sich mit Blick auf die Daseinsvorsorge besonders starke Effekte im Subsample der zwar relativ gut aber doch heterogen versorgten mittelgroßen Gemeinden (5000–20.000 Einwohner) feststellen.^26^ Mit einem R‑Quadrat von über 0,79 weisen die Regressionsoutputs darauf hin, dass ein sehr hoher Anteil der Varianz des AfD-Wahlergebnis auf Gemeindeebene durch die unabhängigen Variablen erklärt wird. Dieser geht aber lediglich zu einem kleinen Teil auf die Variablen der Daseinsvorsorge zurück. Einen besonders hohen Erklärungsgehalt liefern die sozio-demographischen Kontrollvariablen sowie die fixen Effekte auf Bundeslandebene.

Die Kontrollvariablen zeigen größtenteils die erwarteten Effekte. Tatsächlich findet die AfD in Räumen mit niedrigerer Wahlbeteiligung einen fruchtbaren Boden. Der von der Bertelsmann Stiftung (2017) attestierte „AfD-Effekt“ kann somit auch auf Gemeindeebene reproduziert werden. Zu dem beschriebenen gefühlten „Verlassenwerden“ passt auch, dass eine starke Abwanderung der AfD in die Hände zu spielen scheint. Im Vergleich der kleineren Gemeinden geht nichtsdestotrotz ein höherer Anteil älterer Menschen mit einem schwächeren Abschneiden der rechtspopulistischen Partei einher. Zudem schneidet die AfD vergleichsweise besser ab, je höher die Arbeitslosigkeit oder der Anteil von Beschäftigung im Verarbeitenden Gewerbe sind. Insbesondere der Effekt von industrieller Wertschöpfung oder Beschäftigung auf das AfD-Wahlergebnis hat sich in Auswertungen auf Ebene der Landkreise und kreisfreien Städte als robust erwiesen (Bergmann et al. 2018; Manow 2018).^27^ Ein solcher Befund erstaunt, da immer wieder auf die gegenüber dem „Dienstleistungsproletariat“ (Reckwitz 2019, S. 103) positive Lohnentwicklung in der exportorientierten Industrie hingewiesen wird. Trotzdem wird der (noch immer) gutsituierten „alten Mittelklasse“ (Reckwitz 2019, S. 97) ein hohes Maß an Unsicherheit attestiert. Sowohl eine globalisierungs- als auch eine digitalisierungsbedingte Verunsicherung kommen als mögliche Erklärung der entsprechenden politischen Frustration in Frage (Sauer et al. 2018; Manow 2018). Eine solche Interpretation wird durch das Hinzuziehen der IW-Patentdatenbank nach Anmeldersitz gestützt (Koppel und Röben 2019). Da Patentanmeldungen in Deutschland größtenteils auf wenige Industriebranchen zurückgehen, lassen sich die Patentaktivitäten in Kombination mit der Beschäftigung im Verarbeitenden Gewerbe interpretieren. Im Gegensatz zum positiven Zusammenhang zwischen Verarbeitenden Gewerbe und AfD-Wahlergebnis, gehen mehr Patentanmeldungen in einer Gemeinde mit einer geringeren Zustimmung zur AfD einher. Wird die Patentaktivität als Proxy für die Zukunftstauglichkeit und Substituierungsresilienz der lokalen Wirtschaft verstanden, lässt sich interpretieren, dass eine Verunsicherung aufgrund der Abhängigkeit von möglicherweise unsicherer industrieller Fertigung nur insoweit besteht, wie die Betriebe vor Ort tatsächlich eine geringe Innovationstätigkeit aufweisen. Eine innovative Wirtschaft hingegen wäre dann, wie vom Economist (2019) vermutet, als Schutzschild vor rechtspopulistischer Zustimmung zu verstehen.

Dem widerspricht nicht, dass die AfD besser abgeschnitten hat, wo die Arbeitslosigkeit vergleichsweise stark gesunken ist, da diese eben dort vorher ein besonders hohes Niveau aufgewiesen hat. Erstaunlicherweise geht eine höhere Kommunalverschuldung ceteris paribus mit einem schwächeren AfD-Wahlergebnis einher. Dies verwundert angesichts der prominenten AfD-Erfolge in den hochverschuldeten Ruhrgebietsagglomerationen, insbesondere in Sachsen erzielt die AfD jedoch extrem gute Wahlergebnisse in äußerst solventen Gemeinden. An dieser Variable gemessene kommunale Handlungsfähigkeit kann demnach per se keine pazifierende Wirkung bescheinigt werden.

### Europawahl 2019

An der Charakterisierung der Wahlen zum Europäischen Parlament als „second-order national elections“ hat sich seit Reif und Schmitts (1980) Befund nur wenig geändert: Noch immer ähneln die Wahlergebnisse denen auf nationaler Ebene, die Wahlbeteiligung fällt niedriger aus und kleinere Parteien gewinnen tendenziell gegenüber der amtierenden Regierung (Hix und Marsh 2011). Demnach ist es nicht verwunderlich, dass die Regressionsergebnisse zur Europawahl 2019 denjenigen der Bundestagswahl 2017 ähneln. Der ungewichtete Korrelationskoeffizient der Wahlbeteiligungen beider Wahlen liegt bei 0,72; der Korrelationskoeffizient der AfD-Ergebnisse fällt mit 0,94 sogar noch stärker aus.^28^

Im Gegensatz zur Bundestagswahl 2017 hat die Mobilisierung jedoch nicht primär in den gefährdeten Gemeinden stattgefunden, sondern ebenso in den gutsituierten Quartieren der Großstädte (Franz et al. 2019). Trotzdem reproduziert Tab. 2 viele Ergebnisse aus 2017. Eine um eine Standardabweichung intensivere Daseinsvorsorge geht ceteris paribus mit einem um 0,13 Standardabweichungen schlechteren AfD-Wahlergebnis einher.^29^ Für das reduzierte Sample sinkt dieser Effekt auf 0,07 Standardabweichungen. Diese Ergebnisse lassen sich wieder nicht für alle Einzelkategorien reproduzieren: Für die Daseinsvorsorge mit Mobilitätsinfrastruktur zeigt sich sogar ein statistisch signifikantes umgekehrtes Vorzeichen, was auf den entsprechenden Zusammenhang innerhalb der kleinen Gemeinden mit weniger als 5000 Einwohnern zurückgeht.^30^ Dass die Zustimmung zu den Rechtspopulisten ceteris paribus mit steigender Entfernung zum nächstgelegenen Bahnhof sinkt (und eben nicht steigt), und sich gegenteiliges mit Blick auf die Erreichbarkeit des nächsten Krankenhauses zeigt, deutet auf Schwierigkeiten in der Interpretation der suggerierten simplen Zusammenhänge hin.

**Table 2:** 

	(1)	(2)	(3)	(4)	(5)	(6)
	AfD Ergebnis	AfD Ergebnis <20.000	AfD Ergebnis	AfD Ergebnis <20.000	AfD Ergebnis	AfD Ergebnis <20.000
Daseinsvorsorge Index	−0,014*** (0,001)	−0,009*** (0,001)	–	–	–	–
Daseinsvorsorge Mobilität	–	–	0,002*** (0,001)	0,002*** (0,001)	–	–
Daseinsvorsorge Digital	–	–	−0,002*** (0,000)	−0,001 (0,000)	–	–
Daseinsvorsorge Gesundheit	–	–	−0,009*** (0,001)	−0,006*** (0,001)	–	–
Daseinsvorsorge Bildung	–	–	−0,005*** (0,000)	−0,004*** (0,000)	–	–
Distanz Bahnhof	–	–	–	–	−0,001** (0,000)	−0,001*** (0,000)
Erreichbarkeit Krankenhaus	–	–	–	–	0,006*** (0,000)	0,002*** (0,000)
Wahlbeteiligung	−0,119*** (0,005)	−0,089*** (0,005)	−0,128*** (0,005)	−0,100*** (0,005)	−0,112*** (0,005)	−0,083*** (0,005)
Anteil Beschäftigung Verarbeitendes Gewerbe	0,013*** (0,002)	0,002 (0,002)	0,016*** (0,002)	0,004** (0,002)	0,014*** (0,002)	0,001 (0,002)
Einwohnerdichte	−0,008*** (0,001)	−0,000 (0,002)	−0,007*** (0,001)	0,000 (0,002)	−0,014*** (0,001)	−0,012*** (0,002)
Einwohnerdichte^2	0,000*** (0,000)	−0,000 (0,000)	0,000*** (0,000)	−0,000 (0,000)	0,000*** (0,000)	0,000** (0,000)
Anteil Arbeitslose	0,584*** (0,024)	0,406*** (0,027)	0,565*** (0,024)	0,400*** (0,027)	0,528*** (0,024)	0,344*** (0,027)
Veränderung Anteil Arbeitslose	−0,013*** (0,001)	−0,009*** (0,001)	−0,013*** (0,001)	−0,009*** (0,001)	−0,013*** (0,001)	−0,009*** (0,001)
Patentanmeldungen pro Kopf	−0,030 (0,114)	−0,267 (0,267)	−0,082 (0,113)	−0,267 (0,266)	−0,188 (0,115)	−0,420 (0,269)
Altenquotient	0,147*** (0,009)	−0,012 (0,009)	0,163*** (0,009)	0,001 (0,009)	0,119*** (0,009)	−0,023*** (0,009)
Bevölkerungsentwicklung	−0,742*** (0,081)	−0,253*** (0,071)	−0,701*** (0,081)	−0,244*** (0,071)	−1,050*** (0,081)	−0,397*** (0,071)
Kommunalverschuldung	−0,248* (0,141)	−0,514** (0,225)	−0,441*** (0,142)	−0,561** (0,224)	−0,318** (0,144)	−0,817*** (0,225)
Bundesland fixe Effekte	✔	✔	✔	✔	✔	✔
Konstante	0,092*** (0,005)	0,124*** (0,005)	0,094*** (0,005)	0,127*** (0,005)	0,095*** (0,005)	0,127*** (0,005)
*N*	11.049	10.350	11.049	10.350	11.049	10.350
R‑Quadrat	0,823	0,833	0,825	0,834	0,817	0,830

Standardfehler in Klammern

*** *p* < 0,01; ** *p* < 0,05; * *p* < 0,1

Mit Blick auf die Kontrollvariablen fällt im Vergleich zur Bundestagswahl auf, dass im ländlichen Raum eine höhere Zustimmung zur AfD nicht in jeder Spezifikation mit einem höheren Beschäftigungsanteil im Verarbeitenden Gewerbe einhergeht. Zudem steht die Patentaktivität lediglich in den mittelgroßen Gemeinden in einem entsprechenden statistisch signifikanten Zusammenhang mit dem AfD-Wahlergebnis. Damit weisen die beiden untersuchten Wahlen große Ähnlichkeiten auf, zeigen aber ebenso einige wichtige Unterschiede, die sowohl aus der veränderten Anhängerschaft der AfD zwischen 2017 und 2019, als auch aus der unterschiedlichen Zusammensetzung des Elektorates resultieren könnten.

## Diskussion und Zusammenfassung

Der Bevölkerungsschwund vieler ländlicher Kommunen erschwert die Finanzierung einer Daseinsvorsorge, wie diese in den Agglomerationsgebieten angeboten werden kann. Angesichts des Teufelskreises aus „weniger Bevölkerung, weniger Wachstum, weniger Steuern, weniger finanzielle Handlungsmöglichkeiten der öffentlichen Hand“ (Bundesinstitut für Bau‑, Stadt- und Raumforschung 2017, S. 6) sehen viele wissenschaftliche Kommentatoren nur noch den Ausweg einer Bündelung von öffentlichen Mitteln. Das Leibnitz Institut für Wirtschaftsforschung Halle (2019) fordert den Exit aus der Förderung ländlicher Kommunen in Ostdeutschland und einen Fokus auf die wirtschaftsstarken Städte. Die Bildungsministerin signalisiert, dass sie „5G nicht an jeder Milchkanne“ für notwendig hält (Grasnick 2018) und die Bertelsmann Stiftung (2019b, S. 7) fordert „eine stärkere Zentralisierung und Spezialisierung der stationären Versorgung“ von Krankenhäusern. Die enormen finanziellen Anstrengungen aus der Covid-19-Pandemie schlagen sich in Bundes‑, Länder- sowie Kommunalfinanzen nieder und werden die Schwierigkeiten der bereits vorbelasteten Gemeinden weiter verstärken. Dabei zeigt sich schon heute: Selbst wenn etwa in Oberzentren gebündelte Infrastruktur auch Menschen in abgelegenen Regionen eine qualitativ bessere Daseinsvorsorge bieten würde und sogar „gleichwertige Lebensverhältnisse“ (§ 72 GG) besser zu wahren vermochte, bestünde immer die Gefahr der symbolischen Schließung öffentlicher Einrichtungen, die mit einem „Gefühl des Verlassenseins“ (Hillje 2018, S. 2) einhergehen kann.

Studien aus den USA (Cramer 2016), dem Vereinigten Königreich (Essletzbichler et al. 2018; Fetzer 2019; Rodríguez-Pose 2018) und Frankreich (Guilluy 2018; Le Bras 2015) haben zuletzt auf die politischen Implikationen eines solchen Rückzug des Staates hingewiesen. Nachdem sich die Menschen in den *verlassenen* Regionen langsam von der Wahlurne verabschiedet hatten, schlagen die betroffenen Regionen nun in einer „Revenge of Places That Don’t Matter“ (Rodríguez-Pose 2018) durch die Wahl von rechten Parteien außerhalb des politischen Establishments zurück. Auch für Deutschland liegt die Vermutung nahe, in Kommunen mit schlechter Daseinsvorsorge habe eine Entfremdung zu den etablierten Parteien stattgefunden, die sich nun in einer „Geographie der Unzufriedenheit“ (Dijkstra et al. 2019) spiegelt. Tatsächlich hat die lange Zeit sinkende Wahlbeteiligung bei Bundestagswahlen erst mit dem Erfolg der AfD im Jahr 2017 ein vorläufiges Ende gefunden und diesem „AfD-Effekt“ wurde sogar ein Abmildern der sozialen Schieflage der Wahlbeteiligung attestiert (Bertelsmann Stiftung 2017).

Der vorliegende Beitrag eröffnet nun die empirische Analyse des räumlichen Zusammenhangs zwischen disparater öffentlicher Daseinsvorsorge und der politischen Entfremdung zwischen Wählern und etablierten Parteien in Deutschland – insbesondere im ländlichen Raum. Wie erwartet deckt die Regressionsanalyse für die Bundestagswahl 2017 und die Europawahl 2019 einen räumlichen Zusammenhang auf zwischen AfD-Wahlerfolgen und schlechter Daseinsvorsorge, geringer politischer Partizipation, einer angespannten Lage auf dem Arbeitsmarkt sowie Bevölkerungsabwanderung. Grundsätzlich bieten die räumlichen Variablen einen maßgeblichen Erklärungsbeitrag der großen Unterschiede im AfD-Wahlergebnis zwischen deutschen Gemeinden und deuten so auf die Bedeutung regionaler gegenüber den umstrittenen individuellen sozio-ökonomischen Faktoren hin.

Mit Blick auf die Daseinsvorsorge im ländlichen Raum kommt die Analyse hingegen nicht zu dem erwarteten Ergebnis. Zwar sinkt die Daseinsvorsorge klar mit abnehmender Gemeindegröße, sie bietet innerhalb der kleineren Kommunen aber einen wesentlich geringeren oder gar keinen Erklärungsbeitrag zum AfD-Wahlergebnis. Besonders deutliche Effekte lassen sich demgegenüber zwischen den sehr heterogenen mittelgroßen Gemeinden feststellen. Der für Frankreich häufig vorgebrachte Befund, rechtspopulistische Parteien würden mit zunehmendem Abstand zum nächstgelegenen Bahnhof, immer erfolgreicher, lässt sich für Deutschland keinesfalls nachweisen und deutet somit auf die Gefahr der Überinterpretation griffiger aber übersimplifizierender Erklärungsmuster des Niedergangs etablierter Parteien hin. Ebenso wenig kann einer niedrigen Kommunalverschuldung – als Proxy für die Handlungsfähigkeit der Lokalpolitik – eine pazifierende Wirkung nachgewiesen werden. Zudem vermögen auch die vergleichsweise hohen Einkommen aus dem Verarbeitenden Gewerbe den Zulauf zur AfD nicht zu beschränken – ganz im Gegenteil. Dafür hat jedoch eine innovative, zukunftsgerichtete Wirtschaftsstruktur das Potenzial, die Zustimmung zum Rechtspopulismus abzumildern.

Zwar kann damit gezeigt werden, dass die Erfolge der AfD auch im ländlichen Raum geographisch mit wirtschaftlichen Schwierigkeiten einhergehen, um die komplexe Wirkungsrichtung dieser Zusammenhänge zu verstehen und politische Handlungsoptionen auszuloten, bedarf es hingegen tiefgreifenderer und noch kleinteiligerer Analysen. Erst wenn eine breitere Datenbasis zur Daseinsvorsorge über einen längeren Zeithorizont zugänglich ist, die auch qualitative Faktoren umfasst, und ebenso georeferenzierbare Wahlergebnisse auf Wahlbezirksebene vorliegen, kann auseinanderziseliert werden, inwiefern eine schwache oder schwächer werdende Daseinsvorsorge tatsächlich kausal eine politische Entfremdung auslöst oder lediglich räumlich mit spezifisch gewachsenen, kulturellen Einstellungs- und Wertemustern einhergeht. In Verbindung mit Befragungsdaten muss darüber hinaus die Heterogenität der räumlichen Struktur analysiert werden, um zu prüfen, inwiefern Wählerinnen und Wähler in den gefährdeten Regionen tatsächlich von wirtschaftlichen Schwierigkeiten und schwacher Daseinsvorsorge betroffen sind oder ob gerade diejenigen der AfD zuneigen, die entsprechende Probleme in ihrem räumlichen Umfeld beobachten, selbst aber (noch) nicht zu spüren bekommen haben.

### Caption Electronic Supplementary Material





## Anhang

**Table 3:**

Medizinisch	Pkw-Fahrzeit zum nächstgelegenen Krankenhaus
	Anteil Einwohner mit max. 1 km Luftliniendistanz zur nächsten Apotheke
Digital	Anteil Haushalte mit Breitbandversorgung mit 100 Mbit
Verkehrsinfrastrukturell	Pkw-Fahrzeit zur nächsten Autobahnanschlussstelle in Minuten
	Bevölkerungsgewichtete Distanz zum nächstgelegenen DB-Bahnhof
	Anzahl Abfahrten an ÖV-Haltepunkten pro Einwohner
Bildungsinfrastrukturell	Anteil Schüler an allgemeinbildenden Schulen pro Bevölkerung zwischen 6 und 18 Jahren

**Table 4:**

	Mittelwert	Varianz	Std-abweichung	Min	Max
Anteil AfD 2017	0,1372099	0,0047841	0,0691672	0,0069345	0,4501705
Anteil AfD 2019	0,1221816	0,0048783	0,069845	0,0064837	0,4581432
Daseinsvorsorge Index	0	0,3639657	0,6032957	−6,382848	6,973844
Daseinsvorsorge Mobilität	0	0,401107	0,6333301	−6,17979	5,578337
Daseinsvorsorge Digital	0	1	1	−1,593347	1,317035
Daseinsvorsorge Gesundheit	0	0,5852187	0,7649959	−23,00071	1,838484
Daseinsvorsorge Bildung	0	1	1	−0,702555	30,87763
Distanz Bahnhof	0	1	1	−1,341131	8,407843
Erreichbarkeit Krankenhaus	0	1	1	−1,576818	45,20076
Wahlbeteiligung 2017	0,7931178	0,0040042	0,0632786	0,2090839	1
Wahlbeteiligung 2019	0,6615275	0,0080319	0,0896207	0,3959025	1
Anteil Beschäftigung Verarbeitendes Gewerbe	0,1988981	0,0349189	0,186866	0	1
Einwohnerdichte 2017	0,1851858	0,0811313	0,2848355	0,003	4,686
Anteil Arbeitslose 2017	0,0348923	0,0004283	0,0206961	0	0,1942446
Anteil Arbeitslose 2019	0,029566	0,0002763	0,0166224	0,0023346	0,1732852
Veränderung Arbeitslose	−0,1608271	0,1277202	0,3573796	−1	7,043773
Patentanmeldungen pro Kopf	0,0001446	1,26e-06	0,0011246	0	0,0890337
Altenquotient	0,2931182	0,002384	0,048826	0,0948496	0,6666667
Bevölkerungsentwicklung	0,0003767	0,0000308	0,0055463	−0,05	0,21
Kommunalverschuldung	0,0014387	2,74e-06	0,0016551	0	0,0359467

**Table 5:**

	(1)	(2)	(3)	(4)	(5)	(6)
	AfD Ergebnis	AfD Ergebnis <20.000	AfD Ergebnis	AfD Ergebnis <20.000	AfD Ergebnis	AfD Ergebnis <20.000
Daseinsvorsorge Index	−0,141	−0,072	–	–	–	–
Daseinsvorsorge Mobilität	–	–	0,007	0,004	–	–
Daseinsvorsorge Digital	–	–	−0,028	−0,007	–	–
Daseinsvorsorge Gesundheit	–	–	−0,107	−0,055	–	–
Daseinsvorsorge Bildung	–	–	−0,062	−0,047	–	–
Distanz Bahnhof	–	–	–	–	−0,000	−0,002
Erreichbarkeit Krankenhaus	–	–	–	–	0,054	0,015
Wahlbeteiligung	−0,161	−0,126	−0,169	−0,137	−0,139	−0,114
Anteil Beschäftigung Verarbeitendes Gewerbe	0,047	0,014	0,055	0,019	0,051	0,013
Einwohnerdichte	−0,055	0,022	−0,023	0,029	−0,200	−0,033
Einwohnerdichte^2	−0,086	−0,031	−0,099	−0,034	0,030	0,002
Anteil Arbeitslose	0,227	0,100	0,221	0,099	0,215	0,089
Veränderung Anteil Arbeitslose	−0,053	−0,051	−0,052	−0,052	−0,056	−0,050
Patentanmeldungen pro Kopf	−0,010	−0,010	−0,011	−0,010	−0,016	−0,012
Altenquotient	0,059	−0,063	0,069	−0,056	0,038	−0,069
Bevölkerungsentwicklung	−0,033	−0,008	−0,030	−0,007	−0,055	−0,017
Kommunalverschuldung	−0,045	−0,024	−0,069	−0,026	−0,055	−0,031
Bundesland-Dummy	✔	✔	✔	✔	✔	✔
Konstante	0,361	0,437	0,361	0,438	0,363	0,439
*N*	11.049	10.350	11.049	10.350	11.049	10.350
R‑Quadrat	0,798	0,796	0,800	0,797	0,791	0,793

**Table 6:**

	(1)	(2)	(3)	(4)	(5)	(6)
	AfD Ergebnis <5000	AfD Ergebnis: 5000–20.000	AfD Ergebnis <5000	AfD Ergebnis: 5000–20.000	AfD Ergebnis <5000	AfD Ergebnis: 5000–20.000
Daseinsvorsorge Index	−0,006*** (0,001)	−0,015*** (0,001)	–	–	–	–
Daseinsvorsorge Mobilität	–	–	−0,000 (0,001)	0,000 (0,001)	–	–
Daseinsvorsorge Digital	–	–	0,000 (0,000)	−0,001 (0,001)	–	–
Daseinsvorsorge Gesundheit	–	–	−0,004*** (0,001)	−0,010*** (0,001)	–	–
Daseinsvorsorge Bildung	–	–	−0,002*** (0,000)	−0,009*** (0,001)	–	–
Distanz Bahnhof	–	–	–	–	−0,000 (0,000)	−0,000 (0,001)
Erreichbarkeit Krankenhaus	–	–	–	–	0,001 (0,001)	0,005*** (0,001)
Wahlbeteiligung	−0,118*** (0,009)	−0,222*** (0,015)	−0,127*** (0,009)	−0,231*** (0,016)	−0,113*** (0,009)	−0,202*** (0,016)
Anteil Beschäftigung Verarbeitendes Gewerbe	−0,004* (0,002)	0,021*** (0,004)	−0,003 (0,002)	0,023*** (0,004)	−0,005** (0,002)	0,027*** (0,004)
Einwohnerdichte	0,015*** (0,006)	0,004 (0,003)	0,017*** (0,006)	0,004 (0,003)	0,003 (0,005)	−0,006** (0,003)
Einwohnerdichte^2	−0,000*** (0,000)	−0,000 (0,000)	−0,000*** (0,000)	−0,000 (0,000)	−0,000 (0,000)	0,000* (0,000)
Anteil Arbeitslose	0,348*** (0,035)	0,447*** (0,043)	0,353*** (0,035)	0,433*** (0,043)	0,339*** (0,035)	0,405*** (0,044)
Veränderung Anteil Arbeitslose	−0,008*** (0,002)	−0,018*** (0,003)	−0,008*** (0,002)	−0,018*** (0,003)	−0,007*** (0,002)	−0,019*** (0,003)
Patentanmeldungen pro Kopf	−1,148** (0,582)	−0,101 (0,187)	−1,154** (0,581)	−0,134 (0,186)	−1,236** (0,583)	−0,246 (0,190)
Altenquotient	−0,114*** (0,011)	0,090*** (0,017)	−0,108*** (0,011)	0,098*** (0,017)	−0,118*** (0,011)	0,073*** (0,018)
Bevölkerungsentwicklung	−0,025 (0,084)	0,380** (0,177)	−0,012 (0,084)	0,329* (0,176)	−0,055 (0,084)	0,222 (0,180)
Kommunalverschuldung	−2,006*** (0,364)	−0,803*** (0,251)	−1,999*** (0,363)	−0,945*** (0,254)	−2,005*** (0,365)	−0,956*** (0,256)
Bundesland fixe Effekte	✔	✔	✔	✔	✔	✔
Konstante	0,191*** (0,009)	0,210*** (0,014)	0,196*** (0,009)	0,219*** (0,014)	0,190*** (0,009)	0,195*** (0,014)
*N*	8120	2887	8120	2887	8120	2887
R‑Quadrat	0,764	0,824	0,765	0,826	0,763	0,817

Standardfehler in Klammern

*** *p* < 0,01; ** *p* < 0,05; * *p* < 0,1

**Table 7:**

	(1)	(2)	(3)	(4)	(5)	(6)
	AfD Ergebnis	AfD Ergebnis <20.000	AfD Ergebnis	AfD Ergebnis <20.000	AfD Ergebnis	AfD Ergebnis <20.000
Daseinsvorsorge Index	−0,134	−0,073	–	–	–	–
Daseinsvorsorge Mobilität	–	–	0,023	0,016	–	–
Daseinsvorsorge Digital	–	–	−0,031	−0,007	–	–
Daseinsvorsorge Gesundheit	–	–	−0,109	−0,059	–	–
Daseinsvorsorge Bildung	–	–	−0,057	−0,048	–	–
Distanz Bahnhof	–	–	–	–	−0,012	−0,015
Erreichbarkeit Krankenhaus	–	–	–	–	0,066	0,028
Wahlbeteiligung	−0,130	−0,094	−0,140	−0,106	−0,123	−0,088
Anteil Beschäftigung Verarbeitendes Gewerbe	0,033	0,004	0,041	0,009	0,035	0,003
Einwohnerdichte	−0,172	−0,001	−0,147	0,002	−0,301	−0,057
Einwohnerdichte^2	0,098	−0,010	0,092	−0,010	0,201	0,024
Anteil Arbeitslose	0,257	0,101	0,248	0,099	0,232	0,085
Veränderung Anteil Arbeitslose	−0,050	−0,035	−0,048	−0,035	−0,051	−0,034
Patentanmeldungen pro Kopf	−0,001	−0,004	−0,003	−0,004	−0,007	−0,007
Altenquotient	0,108	−0,008	0,119	0,001	0,087	−0,016
Bevölkerungsentwicklung	−0,052	−0,018	−0,049	−0,017	−0,074	−0,028
Kommunalverschuldung	−0,035	−0,011	−0,062	−0,012	−0,045	−0,018
Bundesland-Dummy	✔	✔	✔	✔	✔	✔
Konstante	0,355	0,416	0,356	0,419	0,356	0,417
	11.049	10.350	11.049	10.350	11.049	10.350
R‑Quadrat	0,823	0,833	0,825	0,834	0,817	0,830

**Table 8:**

	(1)	(2)	(3)	(4)	(5)	(6)
	AfD Ergebnis <5000	AfD Ergebnis: 5000–20.000	AfD Ergebnis <5000	AfD Ergebnis: 5000–20.000	AfD Ergebnis <5000	AfD Ergebnis: 5000–20.000
Daseinsvorsorge Index	−0,005*** (0,001)	−0,015*** (0,001)	–	–	–	–
Daseinsvorsorge Mobilität	–	–	0,002*** (0,001)	0,001 (0,001)	–	–
Daseinsvorsorge Digital	–	–	0,000 (0,000)	−0,001 (0,001)	–	–
Daseinsvorsorge Gesundheit	–	–	−0,005*** (0,001)	−0,009*** (0,001)	–	–
Daseinsvorsorge Bildung	–	–	−0,002*** (0,000)	−0,008*** (0,001)	–	–
Distanz Bahnhof	–	–	–	–	−0,001*** (0,000)	−0,001* (0,001)
Erreichbarkeit Krankenhaus	–	–	–	–	0,002*** (0,000)	0,006*** (0,001)
Wahlbeteiligung	−0,080*** (0,006)	−0,141*** (0,009)	−0,091*** (0,006)	−0,144*** (0,010)	−0,079*** (0,006)	−0,141*** (0,010)
Anteil Beschäftigung Verarbeitendes Gewerbe	−0,007*** (0,002)	0,014*** (0,003)	−0,005*** (0,002)	0,017*** (0,003)	−0,008*** (0,002)	0,019*** (0,003)
Einwohnerdichte	0,010** (0,005)	0,000 (0,002)	0,012** (0,005)	−0,000 (0,003)	−0,002 (0,005)	−0,008*** (0,002)
Einwohnerdichte^2	−0,000* (0,000)	0,000 (0,000)	−0,000** (0,000)	0,000 (0,000)	−0,000 (0,000)	0,000*** (0,000)
Anteil Arbeitslose	0,295*** (0,035)	0,591*** (0,042)	0,297*** (0,035)	0,578*** (0,042)	0,268*** (0,035)	0,507*** (0,042)
Veränderung Anteil Arbeitslose	−0,005*** (0,001)	−0,019*** (0,003)	−0,005*** (0,001)	−0,019*** (0,003)	−0,004*** (0,001)	−0,018*** (0,003)
Patentanmeldungen pro Kopf	−1,543*** (0,546)	0,020 (0,171)	−1,541*** (0,544)	−0,022 (0,170)	−1,632*** (0,546)	−0,132 (0,174)
Altenquotient	−0,028*** (0,010)	0,131*** (0,016)	−0,016 (0,010)	0,138*** (0,016)	−0,032*** (0,010)	0,114*** (0,016)
Bevölkerungsentwicklung	−0,081 (0,078)	−0,012 (0,161)	−0,067 (0,078)	−0,064 (0,161)	−0,119 (0,078)	−0,171 (0,164)
Kommunalverschuldung	−1,395*** (0,340)	−0,653*** (0,231)	−1,356*** (0,339)	−0,794*** (0,233)	−1,422*** (0,341)	−0,783*** (0,235)
Bundesland fixe Effekte	✔	✔	✔	✔	✔	✔
Konstante	0,125*** (0,005)	0,105*** (0,009)	0,126*** (0,005)	0,109*** (0,009)	0,127*** (0,005)	0,107*** (0,009)
*N*	8120	2887	8120	2887	8120	2887
R‑Quadrat	0,801	0,856	0,803	0,858	0,801	0,851

Standardfehler in Klammern

*** *p* < 0,01; ** *p* < 0,05; * *p* < 0,1

## Footnotes

1 Siehe INKAR-Datenbank des BBSR: https://www.inkar.de/.

2 Aufgrund der schlechten Datenlage lassen sich die Befunde zur kommunalen Finanzlage nur schwerlich für längere Zeiträume verifizieren. Der Blick auf die kommunalen pro Kopf Ausgaben nordrhein-westfälischer Gemeinden über die vergangenen 25 Jahre zeigt jedoch tatsächlich eine zunehmende Dispersion: Der Variationskoeffizient als Maß der Ungleichheit von pro Kopf Ausgaben steigt von 0,22 im Jahr 1995 auf 0,3 im Jahr 2018 (Siehe Datenbank des Landesbetriebs IT.NRW: https://www.it.nrw/statistik).

3 Einen solchen Zusammenhang hatte in den 1930er-Jahren bereits Tingsten (1975, S. 230) herausgearbeitet: „Je niedriger die Wahlbeteiligung ausfällt, desto ungleicher ist sie“.

4 Mit Blick auf die räumliche politische Entfremdung untersuchen Rösel und Sonnenburg (2016) die Auswirkungen einer Kreisgebietsreform in Mecklenburg-Vorpommern. Für das AfD-Wahlergebnis spielt lediglich die binäre Frage der Ansiedlung von politischen Ämtern eine Rolle, erstaunlicherweise nicht deren tatsächliche Erreichbarkeit.

5 Auch Dorn et al. (2020) betonen die Bedeutung der regionalen Disparitäten der wirtschaftlichen Deprivation als Erklärungsmuster der AfD-Wahlergebnisse, die durchaus den Befunden individueller wirtschaftlicher Betroffenheit entgegenstehen.

6 Allmendinger und Wetzel (2020, S. 49) zeigen, dass eine bessere Erreichbarkeit von Apotheken tatsächlich mit einem intensiveren sozialen Austausch einhergeht.

7 Ein ähnliches Argument findet sich bei Schäfer (2015, S. 165) mit Blick auf Schulen und Vereine.

8 Siehe auch Essletzbichler et al. (2018) für einen ähnlichen Befund.

9 Die Festlegung der noch kleinteiligeren Wahlbezirke obliegt den Gemeinden. Da nur wenige Gemeinden die georeferenzierten Koordinaten der Wahlbezirke veröffentlichen, ist auf der disaggregierten Wahlbezirksebene keine konsistente Analyse im Kontext der regionalen Daseinsvorsorge möglich.

10 Dieses Vorgehen führt zu einer leichten Ungenauigkeit und kann im Einzelfall zu einer berechneten Wahlbeteiligung von über 100 % führen. Um hieraus entstehende unplausible Ergebnisse zu verhindern, wird die Wahlbeteiligung in solchen Fällen mit 100 % angegeben. Problematisch zeigt sich zudem der Kreis Harz in Sachsen-Anhalt, für den dem Bundeswahlleiter lediglich das Gesamtergebnis aller Briefwahlbezirke vorliegt. Auch hier werden die Briefwahlstimmen auf alle zugehörigen Gemeinden verteilt. Interpretationen bezüglich dieses Kreises sollten mit größter Vorsicht betrieben werden.

11 Zwar orientiert sich die Variablenauswahl an in der Literatur als bedeutsam erachteten Dimensionen der Daseinsvorsorge. Die Auswahl ist aber unausweichlich unvollständig und in zukünftigen Forschungsarbeiten zu erweitern und hinterfragen.

12 Die vorliegende Analyse betrachtet Daseinsvorsorge damit quantitativ und nicht wie von der Heinrich-Böll-Stiftung (2017) gefordert nach deren qualitativen Outcomes. Eine „tatsächlich“ qualitative Indikatorik der Daseinsvorsorge auf kommunaler Ebene ist kaum verfügbar, sodass qualitative Indikatoren häufig auf umfragenbasierte subjektive Zufriedenheit zurückgreifen müssen, die nicht unbedingt mit der Qualität der empirischen Daseinsvorsorge übereinstimmen muss, die hier im Vordergrund stehen soll.

13 Die durchschnittliche Erreichbarkeit der Krankenhäuser wurde aus georeferenzierten Daten berechnet: https://www.gkv-kliniksimulator.de/.

14 Als Rohdatengrundlage stellt die deutsche Bahn eine georeferenzierbare Liste der von ihr betriebenen Bahnhöfe zur Verfügung: https://data.deutschebahn.com/dataset/data-stationsdaten.

15 Eine entsprechende Erreichbarkeit in Minuten lässt sich in Ermangelung eines georeferenzierbaren Schulverzeichnis nicht berechnen.

16 Die entsprechenden Daten finden sich hier: https://www.regionalstatistik.de/.

17 Die entsprechenden Daten finden sich hier: https://statistik.arbeitsagentur.de/Navigation/Statistik/Statistik-nach-Themen/Beschaeftigung/Beschaeftigung-Nav.html.

18 Variablen, die das Bildungsniveau der Bevölkerung abbilden, liegen für Deutschland nicht auf Gemeindeebene vor und lassen sich demnach nicht als Kontrollvariablen mit aufnehmen. Das durchschnittliche Bildungsniveau ist aber positiv mit der Gemeindegröße korreliert (Statistisches Bundesamt 2019, Kapitel 3).

19 Der insbesondere für die Konflikthypothese bedeutsame Anteil der ausländischen Bevölkerung wird in Deutschland auf Gemeindeebene nicht valide ausgewiesen.

20 Die entsprechenden Daten finden sich hier: https://www.regionalstatistik.de/.

21 Tab. 3 (im Anhang) gibt einen Überblick über die verwendeten Datenquellen. Tab. 4 (im Anhang) zeigt die deskriptiven Statistiken.

22 Die in Abb. 1 ersichtliche räumliche Struktur der vorliegenden Analyse stellt grundsätzlich in Frage, inwiefern eine OLS-Regression die adäquate ökonometrische Herangehensweise darstellt. Tatsächlich weisen die verwendeten Variablen ebenso wie die Fehlerterme der in Abschn. 4 vorgestellten Regressionen ein statistisch signifikantes Maß räumlicher Autokorrelation (Morans I) auf, sodass OLS-Regressionen verzerrte und ineffiziente Schätzergebnisse liefern. Der Fokus der vorliegenden Analyse liegt jedoch auf dem ländlichen Raum und damit auf einem Subsample deutscher Gemeinden. Die Exklusion der größeren Gemeinden stellt konsistente Regressionsmodelle, die die räumliche Struktur kontrollieren vor grundsätzliche Schwierigkeiten: Einerseits sind es häufig die Großstädte, deren Arbeitsmärkte oder Angebote mit Daseinsvorsorge auf die umliegenden Regionen ausstrahlen, andererseits entstehen im Subsample „künstliche Inseln“ (Kommunen ohne Nachbarschaftsgemeinden) innerhalb Deutschlands, was wiederum Annahmen für die räumliche Gewichtungsmatrix bedingt. Zudem zeigen Maximum Likelihood Schätzer für das gesamte Sample, die die räumliche Struktur kontrollieren (etwa Spatial Simultaneous Autoregressive Error Modelle), qualitativ äquivalente Schätzergebnisse zu den entsprechenden OLS-Regressionen, sodass die vorliegenden Ergebnisse durchaus als eine erste vertiefende Korrelationsanalysen interpretierbar sind. In Zukunft wird die Kontrolle der räumlichen Struktur weiter an Bedeutung gewinnen, sollten Analysen auf Wahlbezirksebene mit noch stärkerer räumlicher Autokorrelation möglich werden.

23 Die Regressionsergebnisse verändern sich qualitativ nicht, wenn anstatt der Bundesland-Dummies der Unterschied zwischen West- und Ostdeutschen Bundesländern in einer einzigen binären Variablen kontrolliert wird.

24 Siehe Tab. 5 (im Anhang) für einen Überblick über die Beta-Koeffizienten der Schätzung in Tab. 1. Der Vergleich und die Interpretation der Effektstärke ist besonders wichtig, da Regressionen mit derart hoher Fallzahl dazu neigen, statistische Signifikanz auszuweisen.

25 Tab. 6 (im Anhang) zeigt, dass die Regressionsergebnisse des Subsamples (<20.000 Einwohner) auch bei einer noch stärkeren Restriktion der Gemeindegrößen (etwa <5000 Einwohner) qualitativ vergleichbar bleiben. Mit Blick auf die Daseinsvorsorge besonders starke Effekte lassen sich zudem im Subsample der mittelgroßen Gemeinden (5000–20.000 Einwohner) feststellen.

26 Siehe Tab. 6 (im Anhang).

27 Tab. 6 (im Anhang) zeigt jedoch, dass Beschäftigung im Verarbeitenden Gewerbe und AfD-Wahlergebnis in kleineren Gemeinden mit weniger als 5000 Einwohnern nicht den hier beschriebenen Zusammenhang aufweist.

28 Aufgrund von Gemeindegebietsreformen lassen sich Wahlbeteiligung und AfD-Wahlergebnis für 253 kleinere Gemeinden nicht eindeutig zuordnen. Die entsprechenden Werte wurden mithilfe von multipler Imputation ergänzt. Die Regressionsergebnisse inklusive dieser Gemeinden unterscheiden sich jedoch qualitativ nicht von den Regressionsergebnissen der 10.794 Gemeinden, für die Wahlergebnisse eindeutig zugeordnet werden können.

29 Siehe Tab. 7 (im Anhang) für einen Überblick über die Beta-Koeffizienten der Schätzung in Tab. 2.

30 Siehe Tab. 8 (im Anhang) für die entsprechenden Regressionsergebnisse mittelgroßer und kleiner Gemeinden.
